# Prospective observational study to assess the performance accuracy of clinical decision rules in children presenting to emergency departments with possible cervical spine injuries: the Study of Neck Injuries in Children (SONIC)

**DOI:** 10.1136/bmjopen-2024-096294

**Published:** 2025-05-02

**Authors:** Natalie Phillips, Geoffrey N Askin, Gavin A Davis, Sharon O’Brien, Meredith L Borland, Amanda Williams, Amit Kochar, Blessy John-Denny, Sarah Watson, Shane George, Michelle Davison, Stuart Dalziel, Eunicia Tan, Shu-Ling Chong, Simon Craig, Arjun Rao, Susan M Donath, Chris J Selman, Stacy Goergen, Catherine L Wilson, Sonia Singh, Nathan Kuppermann, Julie C Leonard, Franz E Babl

**Affiliations:** 1 Emergency Department, Queensland Children’s Hospital, South Brisbane, Queensland, Australia; 2 Biomechanics and Spine Research Group, School of Mechanical, Medical and Process Engineering, Queensland University of Technology, Brisbane, Queensland, Australia; 3 Child Health Research Centre, The University of Queensland, Brisbane, Queensland, Australia; 4 Clinical Sciences, Murdoch Children’s Research Institute, Melbourne, Victoria, Australia; 5 Spinal unit, Department of Orthopaedics, Queensland Children's Hospital, South Brisbane, Queensland, Australia; 6 Emergency Department, Perth Children’s Hospital, Nedlands, Western Australia, Australia; 7 Institute for Paediatric Perioperative Excellence, The University of Western Australia, Perth, Western Australia, Australia; 8 Division of Emergency Medicine, Anaesthesia and Pain Medicine, Medical School, University of Western Australia, Perth, Western Australia, Australia; 9 Divisions of Emergency Medicine, Anaesthesia and Pain Medicine and Paediatrics, Medical School, The University of Western Australia, Perth, Western Australia, Australia; 10 Emergency Department, Women's and Children’s Hospital Adelaide, North Adelaide, South Australia, Australia; 11 Division of Acute Care Medicine, University of Adelaide, Adelaide, South Australia, Australia; 12 Emergency Department, Children’s Hospital at Westmead, Sydney, New South Wales, Australia; 13 Faculty of Medicine and Health, University of Sydney, Sydney, New South Wales, Australia; 14 Emergency Department, Royal Darwin and Palmerston Hospitals, Darwin, Northern Territory, Australia; 15 Emergency Department, Gold Coast University Hospital, Southport, Queensland, Australia; 16 Emergency Department, Sunshine Coast University Hospital, Birtinya, Queensland, Australia; 17 School of Medicine and Dentistry, Sunshine Coast Campus, Griffith University, Birtinya, Queensland, Australia; 18 Caboolture Hospital, Metro North Hospital and Health Care Service, Caboolture, Queensland, Australia; 19 Emergency Department, Starship Children’s Hospital, Auckland, New Zealand; 20 Department of Paediatrics: Child and Youth Health, The University of Auckland, Auckland, New Zealand; 21 Department of Surgery, The University of Auckland Faculty of Medical and Health Sciences, Auckland, New Zealand; 22 Emergency Department, Middlemore Hospital, Auckland, New Zealand; 23 Department of Emergency Medicine, KK Women's and Children’s Hospital, Singapore; 24 Duke-NUS Paediatrics Academic Clinical Programme, Singapore; 25 Paediatric Emergency Department, Monash Medical Centre, Clayton, Victoria, Australia; 26 Department of Paediatrics, School of Clinical Sciences at Monash Health, Monash University, Melbourne, Victoria, Australia; 27 Emergency Department, Sydney Children’s Hospital Randwick, Randwick, New South Wales, Australia; 28 School of Women's and Children’s Health, University of New South Wales, Sydney, New South Wales, Australia; 29 Clinical Epidemiology and Biostatistics Unit, Murdoch Childrens Research Institute, Melbourne, Victoria, Australia; 30 Department of Paediatrics, University of Melbourne, Parkville, Victoria, Australia; 31 Monash Imaging, Monash Health, Clayton, Victoria, Australia; 32 Department of Radiology and Radiological Sciences, School of Clinical Sciences, Monash University, Clayton, Victoria, Australia; 33 Children's National Hospital, Washington, DC, USA; 34 Departments of Pediatrics And Emergency Medicine, The George Washington School of Medicine and Health Sciences, Washington, DC, USA; 35 Division of Emergency Medicine, Department of Pediatrics, The Ohio State University College of Medicine, Columbus, Ohio, USA; 36 Departments of Paediatrics and Critical Care, University of Melbourne, Parkville, Victoria, Australia; 37 Emergency Department, Royal Children’s Hospital, Parkville, Victoria, Australia

**Keywords:** PAEDIATRICS, Emergency Departments, Magnetic Resonance Imaging, Spine, Head & neck imaging

## Abstract

**Introduction:**

Paediatric cervical spine injury (CSI) is uncommon but can have devastating consequences. Many children, however, present to emergency departments (EDs) for the assessment of possible CSI. While imaging can be used to determine the presence of injuries, these tests are not without risks and costs, including exposure to radiation and associated life-time cancer risks. Clinical decision rules (CDRs) to guide imaging decisions exist, although two of the existing rules, the National Emergency X-Radiography Low Risk Criteria and the Canadian C-Spine Rule (CCR), focus on adults and a newly developed paediatric rule from the Pediatric Emergency Care Applied Research Network (PECARN) is yet to be externally validated. This study aims to externally validate these three CDRs in children.

**Methods and analysis:**

This is a multicentre prospective observational study of children younger than 16 years presenting with possible CSI following blunt trauma to 1 of 14 EDs across Australia, New Zealand and Singapore. Data will be collected on presenting features (history, injury mechanism, physical examination findings) and management (diagnostic imaging, admission, interventions, outcomes). The performance accuracy (sensitivity, specificity, negative and positive predictive values) of three existing CDRs in identifying children with study-defined CSIs and the specific CDR defined outcomes will be determined, along with multiple secondary outcomes including CSI epidemiology, investigations and management of possible CSI.

**Ethics and dissemination:**

Ethics approval for the study was received from the Royal Children’s Hospital Melbourne Human Research Ethics Committee in Australia (HREC/69436/RCHM-2020) with additional approvals from the New Zealand Human and Disability Ethics Committee and the SingHealth Centralised Institutional Review Board. Findings will be disseminated through peer-reviewed publications and future management guidelines.

**Trial registration number:**

Registration with the Australian New Zealand Clinical Trials Registry prior to the commencement of participant recruitment (ACTRN12621001050842). 50% of expected patients have been enrolled to date.

**STRENGTHS AND LIMITATIONS OF THIS STUDY**

- This large cohort study will allow for external validation and possible refinement of three existing clinical decision rules on the management of possible cervical spine injuries (CSIs) in children.
- By using a unified study outcome, the study will allow the comparison of the performance accuracy of the three clinical decision rules.
- The study will describe the epidemiology and management of children with potential CSIs across multiple hospitals and countries.
- It is not feasible or ethical to submit all participants to an imaging test to determine the presence or absence of injury. In patients who are not imaged, medical record review and follow-up telephone calls will be used to assess outcomes.
- Using a prospective observational design, the study relies on clinician participation in busy clinical environments for enrolment. Furthermore, it is being conducted within tertiary paediatric and large general emergency departments which may limit its generalisability.

## Introduction

 Paediatric cervical spine injury (CSI) is uncommon, occurring in an estimated 1–2% of paediatric trauma presentations.^1^,^8^ The consequences, however, of such injuries can be devastating, resulting in spinal cord damage, long-term disability and death.^14 9^,^14^ Emergency clinicians therefore seek to identify all CSIs, often through the use of imaging including plain X-ray films (XR), CT scans and MRI.

While paediatric CSI is uncommon, assessment for *possible* CSI is not, and forms part of a standard trauma evaluation in the emergency department (ED). It is unethical and unfeasible to image all children presenting with blunt trauma for possible CSI for a number of reasons including: unnecessary exposure to ionising radiation with associated increased lifetime cancer risk, particularly with CT scans^15^,^21^; the risks of pharmacological sedation often required for young or uncooperative patients with some imaging modalities^22^,^24^; resource implications (cost, time, bed space)*;* and patient discomfort and potential harm with prolonged assessments and the use of spinal motion restriction techniques or ‘immobilisation’.^25^,^28^ Clinicians are thus faced with the decision of which children require imaging, and in whom it can be safely avoided. High imaging rates relative to the number of injuries detected have been reported from multiple countries.^5 8 29^

To address these concerns, attempts have been made to risk stratify patients with blunt trauma and identify those patients at higher risk of CSI, and thus in need of imaging, through the use of clinical decision rules (CDRs). CDRs are composed of at least three variables of patient history, physical examination findings or simple tests, and assist clinicians in making diagnostic or therapeutic decisions. CDR development is a three-step process involving derivation, validation and impact analysis (assessing the rule effect on clinician behaviour). CDRs also require validation in different population groups from which they were derived, with clinicians at different centres to ensure their wider applicability and to validate their findings,^30^,^32^ a process better known as external validation.

Until recently, there was limited paediatric-specific evidence to guide this decision-making process in the assessment of children with possible CSIs. Two high-quality and well-established CDRs, the US National Emergency X-Radiography Low Risk Criteria (NEXUS)^33 34^ and the Canadian C-Spine Rule (CCR),^35^ have been derived primarily, or entirely in adult cohorts. The features of each are detailed in table 1. Based on large prospective datasets, both CDRs are highly sensitive and efficient in reducing excess cervical spine imaging in adults. In the absence of well-validated paediatric-specific decision tools, these rules have often been used in paediatric settings, either in combination or with individual practitioner or institutional modification.^36^,^39^ The validity of their use in children, however, has been heavily questioned, particularly at younger ages.^3 40 41^ The CCR has not been validated in a paediatric cohort, and NEXUS, while reporting 100% sensitivity in their paediatric cohort, had a median paediatric age of 15 years and included only four children with CSIs younger than 9 years. Some studies applying rule criteria retrospectively have found neither performs well enough for use in children younger than 8 years.^3 40^ Two successive Cochrane reviews by Slaar *et al* and Tavender *et al*^41 42^ have identified similar issues with the application of these CDRs to paediatric populations, advising that large multicentre studies are needed to assess rule performance and projected effects on imaging rates, with particular consideration given to younger cohorts.

**Table 1:** Comparison of NEXUS,^34^ CCR^35^ and PECARN^44^ criteria: study type, ages, inclusion criteria and ‘rule’ features

	NEXUS	CCR	PECARN
Study type	Prospective, observational	Prospective, observational	Prospective, observational
Age	All ages	≥16 years	<18 years
Numbers	3065 children (<18), median age 15 years34 069 patients in total (adults and children)	No children8924 enrolled	22, 430(11 857 in validation cohort, 10 572 in derivation cohort)
Inclusion criteria	Radiographic evaluationBlunt trauma	**GCS 15 and stable**Neck pain from any mechanism or, all of—visible injury above clavicles, non-ambulatory and dangerous mechanism of injury*Exclusion:* ***penetrating trauma****, known vertebral disease, acute paralysis, injury>48 hours, representation, pregnant*	Known or suspected exposure to blunt trauma with at least one of the following: evaluation by a trauma team; transported from the scene of injury to the participating hospital by emergency medical services; underwent cervical spine imaging at the participating hospital, or before transfer to the participating hospital*Exclusion: solely* ***penetrating trauma***
Rule features: history		Age>65 years	
Rule features: mechanism		Dangerous mechanism:Fall≥1 m or five stairsAxial load, for example, divingMVC>100 km/hour, rollover or ejection from vehicle; MVC involving recreational vehicleBicycle collision.	
Rule features: examination	**Altered mental status**GCS<15, disorientated, impaired memory, inappropriate response to external stimuli**IntoxicationFocal neurology**Examiner elicited or patient reported**Painful distracting injury**Any condition thought by the clinician to be producing pain sufficient to distract the patient from a CSI, for example, any long bone fracture, a significant visceral injury, a large laceration, degloving injury or crush injury, extensive burns, any other injury producing acute functional impairment**Posterior midline neck tenderness**	**GCS<15, unstable** → *exclusion criteria***Paraesthesia in extremities*Absence of low risk factor* enabling neck movement to be assessed**.*Low risk factors include:No **midline cervical spine tenderness**Delayed onset of neck painSitting position in EDSimple rear end MVCAmbulatory at any time*Inability to actively rotate neck45 degrees left and right (*providing able to be assessed—see above*)	**Altered mental status** GCS 3–8 or unresponsive on the AVPU scale (Alert, Voice, Pain, Unresponsive)* Altered mental status: defined as GCS score of 9–14; verbal or pain on AVPU; or other signs of altered mental status. Focal neurological deficits on examination*(includes paraesthesia, numbness or weakness)Abnormal airway, breathing or circulation*Substantial head or torso injurySubstantial injuries were defined as those that warranted inpatient observation or surgical intervention**Self-reported neck pain or neck tenderness on examination**
Rule guidance	If no criteria present, considered at very low probability of clinically significant CSI and imaging is not required.	Provides a flow chart with specific guidance on when imaging and no imaging is indicated. Imaging is not indicated if there are no high-risk factors, a low-risk factor enabling neck movement to be assessed is present AND neck movement is assessed as normal.	If high-risk features present (*)—consider CTIf other risk factors present on examination—consider plain X-rayIf no risk factors present—consider clinical clearance without imaging

CCR, Canadian C-Spine Rule; CSI, cervical spine injury; ED, emergency department; GCS, Glasgow Coma Scale; MVC, motor-vehicle crash; NEXUS, National Emergency X-Radiography Low Risk Criteria; PECARN, Pediatric Emergency Care Applied Research Network.

The Pediatric Emergency Care Applied Research Network (PECARN) sought to address the paucity of paediatric specific CSI CDRs, systematically developing a paediatric CSI risk assessment tool.^5 43 44^ A retrospective case control study (540 children with CSI across 17 centres) identified eight CSI-associated variables,^43^ and a subsequent prospective pilot study of 4000 children assessed the performance of the retrospective and a de novo model.^5^ Most recently, a study of 22 430 children derived and validated a CDR^44^ with a reported sensitivity of 94.3% (95% CI 90.7% to 97.9%), a specificity of 60.4% (95% CI 59.4% to 61.3%) and a negative predictive value of 99.9% (95% CI 99.8% to 100%). However, this rule is yet to be externally validated.

In addition to identifying all serious injuries, imaging-related CDRs also often aim to safely reduce the use of imaging. This is particularly important in children where ionising radiation exposure has been associated with increased lifetime cancer risk.^15^,^1719^ In an Australian single-centre study, strict application of NEXUS, CCR and retrospectively derived PECARN criteria would have increased imaging rates.^45^ This unintended consequence of the application of CDRs has been noted previously, with the desired high sensitivity often achieved at the expense of specificity.^46^,^48^ Understanding how different CDRs perform in varied clinical environments, and how they compare with current practice, highlights the importance of external validation.

NEXUS and CCR CDRs do not address the other pressing question of paediatric cervical spine imaging, namely, not only who we should image but which imaging modality should be used and when. Different imaging modalities carry different risks and costs and have different reported sensitivities in detecting CSI. Risk stratification in the PECARN rule provides such guidance with higher risk criteria triggering a CT scan and lower risk criteria triggering plain radiography of the cervical spine.^44^

The primary aim of the ‘Study of Neck Injuries in Children (SONIC)’ is to externally validate existing CDRs (PECARN, NEXUS, CCR) in an international paediatric cohort, assessing their performance accuracy and the potential impact of these rules on imaging utilisation and injury detection. This study will also enable an assessment of the epidemiology and management of possible and confirmed paediatric CSI, and missed injury rates, across study hospitals and countries. Depending on the accuracy of the validation of the existing rules, there is the possibility of refining an existing or deriving a new clinical decision rule to improve accurate detection of CSI and/or risk stratification of children with suspected CSI.

## Methods and analysis

### Design

This is a multicentre multinational prospective observational study. Study reporting will follow the Strengthening the Reporting of Observational Studies in Epidemiology,^49^ the Standards for Reporting Diagnostic Accuracy^50^ and where relevant, the Standard Protocol Items: Recommendations for Interventional Trials^51^ guidelines. This study has been registered with the Australian New Zealand Clinical Trials Registry (ACTRN12621001050842).

### Setting

A total of 14 hospitals are participating in this study: 13 within the Paediatric Research in Emergency Departments International Collaborative in Australia and New Zealand and the KK Women’s and Children’s Hospital, Singapore. Sites and hospital type are listed in table 2.

**Table 2:** Participating sites

Country	Participating hospital emergency departments	Type of hospital
Australia	Royal Children’s Hospital, Victoria	Paediatric
	Queensland Children’s Hospital, Queensland	Paediatric
	Perth Children’s Hospital, Western Australia	Paediatric
	Children’s Hospital at Westmead, New South Wales	Paediatric
	Women’s and Children’s Hospital, South Australia	Paediatric
	Sydney Children’s Hospital Randwick, New South Wales	Paediatric
	Monash Children’s Hospital, Victoria	Paediatric
	Gold Coast University Hospital, Queensland	Adult and paediatric
	Logan Hospital, Queensland	Adult and paediatric
	Royal Darwin Hospital, Northern Territory	Adult and paediatric
	Sunshine Coast University Hospital, Queensland	Adult and paediatric
New Zealand	Starship Children’s Hospital, Auckland	Paediatric
	KidzFirst Hospital, Auckland	Adult and paediatric
Singapore	KK Women’s and Children’s Hospital	Paediatric

### Inclusion criteria

Children aged younger than 16 years with possible CSI after known or suspected blunt trauma.

Possible CSI is defined as (1) initiation of spinal precautions pre-arrival, or (2) neck pain, or (3) considered at risk of CSI by any ED assessing clinician, in the context of blunt trauma.

### Exclusion criteria

There are no specific exclusion criteria. However, patients who receive cervical spine imaging at a centre not participating in the study prior to transfer with external radiology reporting available at the time of arrival at the study ED and patients with solely penetrating trauma will be excluded from the accuracy analysis components of the study.

### Outcomes

#### Primary outcome

Performance accuracy (sensitivity, specificity, negative predictive value (NPV) and positive predictive value (PPV)) in identifying the study defined CSI (table 3) of

1. The PECARN risk criteria,^44^
2. The two adult-derived CDRs (NEXUS and CCR) and
3. Current CSI management practice.

**Table 3:** Definitions

Study defined CSI	Cervical spine injury (CSI) is defined as vertebral fracture, ligamentous injury, intraspinal haemorrhage or spinal cord injury (SCI as diagnosed on MRI or SCI without radiological association) of the cervical region of the spine (occiput to seventh vertebra including ligamentous structures attaching seventh vertebra to first thoracic vertebra).The presence of CSI will be determined by review of the study site cervical spine imaging reports and if applicable, spine surgeon consultation notes and phone follow-up.
Imaging confirmed CSI	Formal radiology report of any trauma-related radiological cervical spine abnormality on plain radiography, CT or MRI scan.This includes vertebral fracture, facet joint subluxation or dislocation, ligamentous injury, disc injury, intraspinal haemorrhage (including subdural and extradural haemorrhage in the spinal canal) and spinal cord injury of the cervical region of the spine (occiput to seventh cervical vertebra including ligamentous structures attaching seventh vertebra to first thoracic vertebra, and C7-T1 disc).Formal imaging reports (ie, reports reviewed and finalised by fully qualified (consultant) radiologists) will be used to determine radiological diagnosis. If the diagnosis on the imaging report conflicts with the spinal surgeon consultation notes, the treating spinal surgeon will be contacted for clarification. For discrepancies between multiple imaging modalities, the interpretation of CT and MRI will supersede the interpretation of plain radiographs.
Clinically important CSI	(1) Death where CSI could be a contributing factor, (2) the need for surgical intervention for CSI, (3) any CSI-related neurological abnormality lasting>7 days or (4) imaging confirmed CSI treated with cervical spine immobilisation lasting>7 days.
Possible CSI	(1) Initiation of spinal precautions pre-arrival (2) neck pain, or (3) considered at risk of CSI by any assessing clinician, in the context of blunt trauma

#### Secondary outcomes

1. Performance accuracy (sensitivity, specificity, NPV and PPV) in identifying *clinically important CSI* (table 3) of (1) the PECARN risk criteria, (2) the two adult-derived CDRs (NEXUS and CCR), (3) current CSI management practice and (4) any newly developed exploratory SONIC CDR.
2. Performance accuracy (sensitivity, specificity, NPV and PPV) in identifying CSI as defined by existing CDRs or risk criteria of (1) the PECARN risk criteria, (2) the two adult-derived CDRs (NEXUS and CCR), (3) current CSI management practice and (4) any newly developed exploratory SONIC CDR.
3. Performance accuracy (sensitivity, specificity, NPV, PPV) in identifying patients with *study-defined CSI* with any newly developed exploratory SONIC CDR.
4. Rates of study defined CSI; imaging-confirmed CSI; clinically important CSI; CSI-related neurological abnormality
5. Rate of surgical intervention of the cervical spine for CSI
6. Determination of missed CSI rates, if any, with different cervical spine imaging modalities (XR vs CT vs MRI)
7. Methods of, duration of and adverse events associated with cervical spine immobilisation
8. Cost effectiveness of CDRs and different imaging modalities compared with usual clinical care.
9. Epidemiology of CSI in participating countries.

### Definitions

See table 3.

### Patient recruitment, study procedures and data collection (flow chart, data collected)

All patients presenting to participating EDs will be screened for eligibility. Treating clinicians will enrol eligible patients. Identification of potentially eligible and missed eligible patients will be undertaken by the research team in each participating centre through a review of ED attendance records. Limited de-identified data will be collected on eligible patients deemed as missed, including any imaging undertaken, and the presence or absence of CSI.

**Figure 1:**
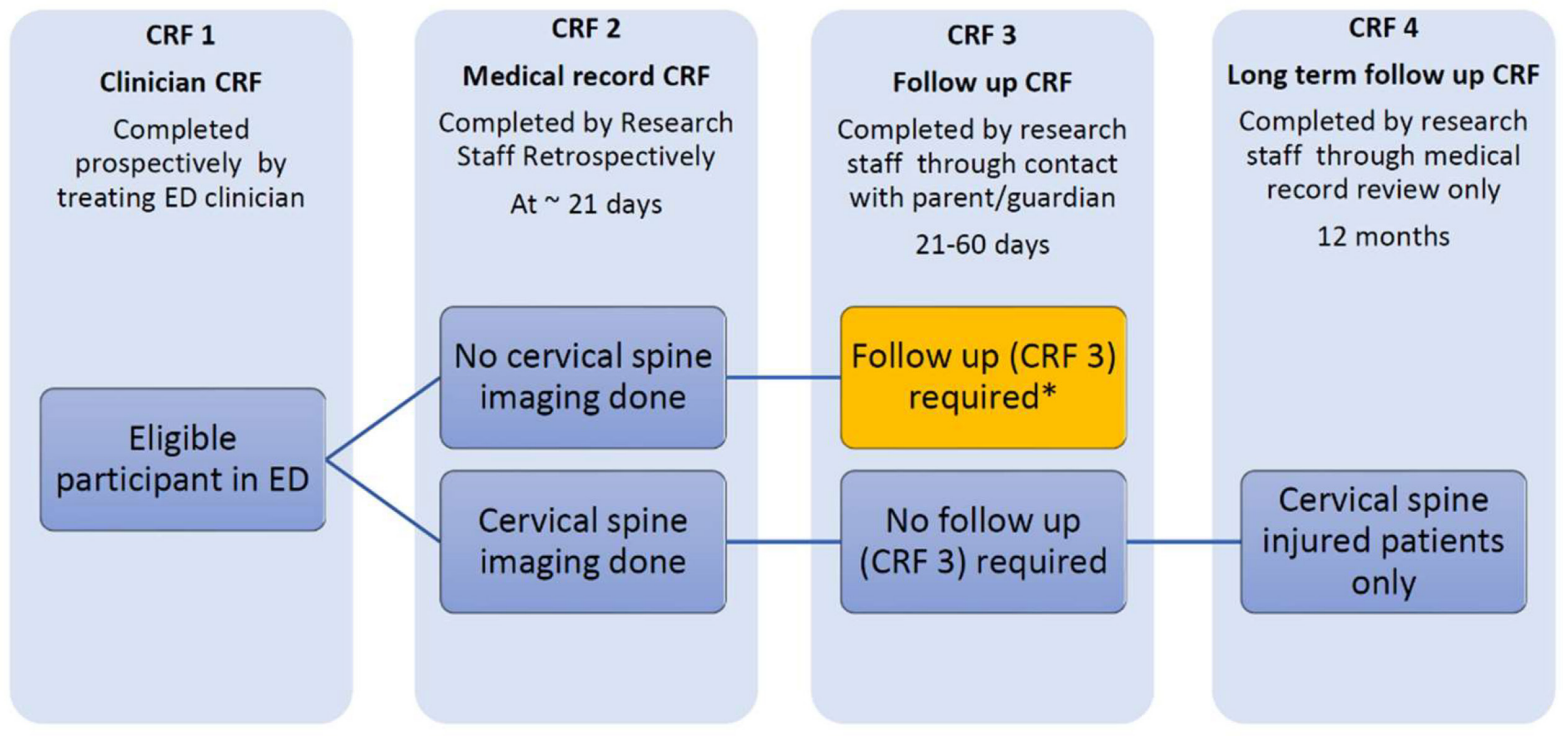
Study flow. * Verbal consent for follow up required (CRF 3). CRF, case report form; ED, emergency department.

Case report forms (CRFs) will be used to collect relevant data at separate time points (figure 1).

The clinician report form (CRF 1) will be completed by the treating clinician at the time of the ED visit. Data collected will include all possible predictor variables in the PECARN risk criteria^44^ and existing adult focused CDRs^32^,^35^ prior to management decisions. Additional information to be collected is detailed in table 4. Clinical management will proceed independent of study participation.

**Table 4:** Data variables to be collected

Clinical report form (CRF)	Variables collected
Clinician report form (CRF 1)	All predictor variables for CDRs under consideration.Mechanism of injury, history of injury (including symptoms and signs prehospital) and clinical ED examination.Clinician perceived CSI likelihood, neck immobilisation practice, planned imaging.
Medical record review (CRF 2)	Detailed demographics; prehospital management; management and imaging undertaken elsewhere for this injury; time-related data (times of triage, clinician evaluation, ED and hospital discharge); duration of ED and hospital stay; admission status; specialty unit consultations; use, type and duration of spinal immobilisation; analgesia; observation duration; intensive care admission, need for intubation and ventilation, duration of ventilation and duration of intensive care unit admission; specialty unit follow-up; any neck injury related representations (and imaging and interventions arranged or completed at that time); all head and neck imaging and results; CSI-related and head-injury related surgical interventions; other significant injury and mortality.
Follow-up contact (CRF 3)	CSI related symptoms (including neck pain, limb weakness/sensory changes/paraesthesias); relevant health practitioner contacts; need for and duration of ED and hospital admissions (with review of the relevant medical record); use, type and duration of spinal precautions and neurological interventions; head and cervical spine imaging modalities and results; CSI-related and head injury-related surgical interventions.
Medical record review for patients with CSI at 12 months (CRF 4)	Imaging, interventions and outcomes at 12 months in patients with confirmed CSI.

CDRs, clinical decision rules; CSI, cervical spine injury; ED, emergency department.

A detailed medical record review (CRF 2) will be completed on or after 21 days after injury by the site research team, collecting information on patient demographics, management and outcomes (table 4).

Follow-up contact (CRF 3) by the site research team will be undertaken on patients who do not receive cervical spine imaging during their initial ED visit or on subsequent attendances (as identified by medical record review at 21 days). This follow-up is undertaken to minimise the potential for missed CSIs, as it is both unethical and unfeasible to image all children presenting with possible CSI due to concerns with exposure to ionising radiation, and healthcare resource use. Follow-up contact will occur 21–60 days after the injury via telephone or email (maximum of six attempts). If more than 60 days have elapsed, or there are six failed contact attempts, follow-up will be regarded as unsuccessful. The medical record for patients unable to be reached will be reviewed for injury-related attendances.

Patients with confirmed CSI who received ongoing follow-up at the hospital after the ED visit will have a retrospective review of the site medical record at twelve months to examine imaging, interventions and outcomes in this subgroup.

Verbal consent for follow-up contact will be obtained at the time of the initial ED visit or at parent/guardian contact during the 21–60 day follow-up period. A waiver of consent has been obtained for medical record review.

### Data management

Data for this study will be collected and entered using paper-based (CRF 1) and electronic data collection forms (CRFs 2–4) which will be completed by the treating clinician and research team, respectively. Participating clinicians (physicians and nurse practitioners) and the research team at all sites receive formal training prior to the commencement of the study. Standardised teaching materials have been created and provided to participating sites and a source document plan has been completed at each site to ensure data consistency.

All data will be de-identified and entered into a secure password-protected database enabled through the REDCap^51^ (Research electronic Data Capture) web-based application hosted by the Murdoch Children’s Research Institute, Melbourne, Australia. All data transmissions are encrypted. This database will only be accessible to trained research staff, with individual site research teams only having access to their own site’s data. Hard copy data will be stored in a secure location accessible only to the local research team. All sites will maintain a separate password-protected logbook, accessible only to local site staff on a secure online database containing reidentifying information for data queries and patient follow-up.

Recruitment, study process, data collection and data entry compliance will be regularly reviewed remotely and through in-person site visits as outlined in a study clinical monitoring plan. Monthly recruitment reports will be undertaken, and individual site support provided by the central coordinating team as required.

All data will be retained in line with ethics and governance requirements of the local site. An established study steering committee comprised of the central study team, local site principal investigators, spinal surgeons, statisticians and international experts will oversee study execution.

### Statistical methods

Statistical analyses will be performed once all data have been collected (with no plan for an interim analysis) using statistical software Stata IC 18.0^52^ or higher.

Demographic data and other relevant variables related to the management of CSI (eg, medical history, injury mechanism, mental status, physical examination) will be summarised as means and SD for continuous data (or medians and IQRs if skewed), or counts and percentages for categorical data, for all enrolled participants.

#### Accuracy analysis

All patients eligible for accuracy analysis will be used to externally assess performance accuracy (sensitivity, specificity, NPV and PPV) in identifying study-defined CSI, imaging-confirmed CSI and clinically important CSI of the PECARN risk criteria,^44^ and NEXUS^34^ and CCR^35^ CDRs. When applying each CDR, items will be scored as present, absent or unknown. ‘Unknown’ predictor variables will be imputed as ‘rule negative’. The estimates of each performance accuracy measure will be reported with exact binomial 95% CIs. A sensitivity analysis will be conducted for all accuracy outcomes by using available data only, treating unknown predictor variables as missing unless one of the completed predictor variables is already rule positive. An additional sensitivity analysis for all accuracy outcomes will include transferred patients who received cervical spine imaging with radiology reports at a non-participating centre. A subgroup analysis will be conducted that restricts the accuracy analysis for patients younger than 9 years and those 9 years and older.

The accuracy of each CDR will also be calculated using the exact inclusion, exclusions, predictor and outcome variables and definitions as set out in the original publications.^34 35 44^

#### Analysis of non-accuracy related outcomes

The entire data set that meets the definition of the study population (ie, those patients that meet all inclusion criteria and do not meet any exclusion criteria) will be used to analyse all other secondary outcomes not related to accuracy using only available data. The risk of study-defined, imaging-confirmed, clinically important CSI, CSI-related neurological abnormality and surgical intervention of the cervical spine, as well as methods of and adverse events associated with cervical spine immobilisation, will be estimated and reported with exact binomial 95% CIs. Duration of cervical spine immobilisation among those without injuries will be reported with median time and corresponding 95% CI. Determination of missed study-confirmed CSI rates with different cervical spine imaging modalities will be compared between each modality (XR, CT, MRI) by estimating a risk difference reported with a 95% CI and p value, using binomial regression. Bivariate associations between epidemiological factors (such as demographics, injury mechanism, medical history, mental status, physical examination) and cervical spine injury will be compared using a risk (binary outcome) or mean difference (continuous outcome) between those with and without cervical spine injury, estimated using either a binomial or linear regression model. For continuous outcome data that appear skewed (such as hospital length of stay), the data will be transformed on the log scale and groups will be compared using log-mean differences. All comparisons will also be reported with 95% CIs and p values.

There may be missing data in some secondary objectives that include the entire eligible sample (ie, non-accuracy related objectives). As such, we will conduct a sensitivity analysis where multiple imputation using chained equations will be used to handle the missing data under a plausible missingness assumption, if appropriate, when there is>5% missingness in key variables in the statistical model to assess the robustness of results.

#### Derivation and validation of a SONIC CDR

Depending on the accuracy of the existing CDRs, we may conduct an exploratory analysis of the data to determine whether a new paediatric SONIC CDR should be derived and validated, or an existing CDR refined, to improve the accuracy of CSI detection and/or improve risk stratification of children who do and do not require imaging. The details of this exploratory analysis will be detailed in a separate protocol.

#### Sample size

The sample size has been calculated based on a secondary outcome (derivation and validation of a new exploratory SONIC CDR), as the sample size required for this will be higher than for the primary outcome. Although not based on the primary outcome, given we expect more events based on this sample size than if the study were powered on the primary outcome, we consequently expect considerable certainty in the estimates of the performance accuracy measures. Based on the prospective pilot study of~1000 children with suspected CSI with~0.5% CSI,^45^ we expect to be able to enrol up to 30 000 patients at 14 sites over 3 years. A high enrolment rate and low loss to follow-up rate is expected based on experience in a similar study.^47^

We will continuously grow the cohort and use all the patients enrolled at the time that 100 CSIs have been identified to derive the rule, and the additional cohort when an extra 50 CSIs have been enrolled to validate the resulting rule. If the rate of CSI is higher than expected, we will be able to reduce the total number of enrolled patients for derivation and validation cohorts. A retrospective pilot study at one of the study hospitals^53^ (CSI rate of 4%) indicated that the expected CSI rate may be higher, which would either increase the precision of the results or reduce the required total number of enrolled patients. The total patient number needed will therefore be adjusted as the study progresses. The CSI rate among enrolled participants to date is higher than the assumptions used to calculate the sample size.

### Economic evaluation

While a cost-effectiveness analysis of CDRs for the diagnosis of CSI in adults has been published by the National Institute for Health and Care Excellence, there have been no prior assessments for children due to lack of data.^54^ A decision analytical model will be constructed from the Australian publicly funded healthcare system perspective with two horizon timelines (acute and long-term). The acute care time horizon will include ED presentation and hospitalisation. The cost-effectiveness analyses will compare the incremental cost-effectiveness ratios of the existing CDRs combined with different imaging modalities to detect significant CSI in children. SONIC study data will be used to determine imaging rates and children’s short-term outcomes. Costs associated with ED presentation, imaging and ongoing clinical care will be incorporated.

The average cost of ED presentations will be derived from the relevant Australian Emergency Care classification.^55^ The costs of imaging will be obtained from the Medicare Benefits Schedule.^56 57^ Direct healthcare costs associated with acute hospitalisations will be estimated using activity-based funding estimates provided by the Independent Health and Aged Care Pricing Authority.^58^ The acute care costs associated with hospitalisation for spinal cord injury will be derived from the National Efficient Price adjusted by the national weighted activity units for the price weights associated with the relevant Australian Refined Diagnosis Related Groups codes. Price weight adjustments are made for paediatrics, care in specialised children’s hospitals, residential remoteness, intensive care, and ventilatory support.

Long-term outcomes following spinal cord and neck injury will be estimated from the subset of children with study-defined CSI whose medical records will be tracked to 12 months and quality of life/utility values will be applied to these health states using estimates from the published literature.^59^ The ongoing hospitalisation costs associated with rehabilitation will be estimated from the paediatric specific price weights for spinal cord injury.^58^ The risk, cost and quality of life impacts of cancer resulting from imaging radiation exposure will be estimated based on published epidemiological studies of risk. Results will be presented as an incremental cost per quality adjusted life year (QALY) loss and net monetary benefit using a threshold of $A50 000 for each CDR compared with usual practice. A discount rate of 5% will be applied to QALYs and future costs associated with spinal cord injury. Probabilistic sensitivity analyses will assess the robustness of the models and the uncertainty of the key input parameters.

### Ethical issues, consent and dissemination

Central ethics approval for the study was received from the Royal Children’s Hospital Melbourne Human Research Ethics Committee in Australia (HREC/69436/RCHM-2020) under the National Mutual Agreement, with New Zealand sites and the KK Hospital in Singapore receiving additional local ethics approval (New Zealand Human and Disability Ethics Committee (HDEC 2022: 11325); SingHealth Centralised Institutional Review Board (E ref: 2021:2401)). All participating sites obtained appropriate individual institutional governance and regulatory approvals as required. The Murdoch Children’s Research Institute in Melbourne, Australia, is the primary sponsor for this trial.

Consent requirements vary across national jurisdictions. For Australian sites, a waiver of consent has been approved for enrolment in the study, and completion of ED clinician and medical record report forms, given information sought constitutes part of routine clinical care. Verbal consent for follow-up contact will be sought from parents of children/adolescents presenting who require such contact. The verbal consent record tool, and parent/carer information sheet are in online supplemental material 1. For New Zealand sites, verbal consent will be sought for all participants. A regulatory waiver of consent has been granted for Singapore. A waiver of consent has also been granted for all patients who are severely injured or die (most would have had cervical spine imaging), given the study is observational in nature and the data is obtained from routine clinical care.

### Risk management, adverse events and patient safety

As this is an observational study, there are no significant envisaged risks to participants. Care is provided by the treating healthcare team independent of the study. Any medical issues identified by research staff will be referred to managing clinicians (for patients in hospital) or site investigators (for discharged patients) who will refer the patient for appropriate clinical review.

Loss of privacy or accidental disclosure of personal information is a potential risk in this study, although this risk is deemed to be low with only de-identified data transmitted to the central site. Study procedures and data storage and protection measures are designed to minimise this risk.

### Patient and public involvement

We did not include patients or families in the design of the study.

### Time plan

The study commenced in September 2021 and is expected to be complete by August 2026.

## Discussion

This study aims to externally validate three existing CDRs used in the assessment of possible paediatric CSI. It will allow the determination of the comparative accuracy of the three rules using identical outcome measures as well as the three rule-specific outcomes. This will expand the depth of knowledge on this topic, enhancing the robustness of evidence available to clinicians worldwide to guide decisions for a common emergency presentation and thus potentially reduce harm to children and costs to healthcare systems by better targeting neck imaging. As the largest paediatric prospective dataset across three countries and outside of North America, it will improve epidemiological information on neck injuries in study countries. Furthermore, given the size of our study, we have the potential to refine existing guidance or derive and validate a new CDR to compare with the others.

## Supplementary material

10.1136/bmjopen-2024-096294 online supplemental file 1
