# Supplementary material for: Prospective observational study to assess the performance accuracy of clinical decision rules in children presenting to emergency departments with possible cervical spine injuries: the Study of Neck Injuries in Children (SONIC)
Source: BMJ Open. 2025 May 2;15(5):e096294. doi: 10.1136/bmjopen-2024-096294 (PMC12049922; doi:10.1136/bmjopen-2024-096294)
Supplement: online supplemental file 1 [file bmjopen-15-5-s001.pdf]

PREDICT Paediatric Research in Emergency Departments International Collaborative

SONIC
Study of Neck Injury in Children

Example of Verbal Consent documentation (on the Clinician Case report form). Australian Version.

IF NO IMAGING WAS DONE & CLINICALLY APPROPRIATE:

Please request verbal consent from the parent/guardian for the study team to contact them via their preferred method at 21-60 days to ensure there are no further concerns of neck injury.

Please give the family the patient information form.

Verbal consent for follow up

1. Study Info sheet given Y ☐ N ☐
2. Verbal Consent (***choose an option***)

A. Consent for follow up obtained ☐

Parent/guardian name: _______________

Best contact (mobile/email): ___________

B. Declined follow up, consented to data use ☐

Declined follow up **and** declined data use ☐

Reason for declining if known____________

C. Consent not obtained - forgot to ask ☐

- Parents not present ☐
- Other: ___________ ☐

PREDICT Paediatric Research in Emergency Departments International Collaborative

SONIC Study of Neck Injury in Children

Insert HEADER with institution's name or institution's letterhead

Parent/Guardian Information Form

**HREC Project Number:**	*69436*
**Full Name of Project:**	SONIC – Study of Neck Injuries in Children
**Principal Researcher:**	*<Insert site PI>*
**Version Number and Date:**	Version 3, 19 May 2023

The Emergency Department at *<insert local hospital>* is conducting a review of all children who present after a possible neck injury. We would like to collect some information about every one of these children, including your child.

This study has been approved by The Royal Children's Hospital Human Research Ethics Committee and we ask you to consider your child's participation in this study. You and your child do not need to do anything special for this study and involvement will only take a few minutes of your time. Your child's care in the emergency department and within the hospital will not change.

This project is part of a national study on children who are seen in the emergency department with a possible neck injury. The study is being conducted at 13 major hospitals in Australia, New Zealand and Singapore, and is funded by an Australian Government grant.

As part of this study, we want to get a better understanding of which children attend hospital with possible neck injuries. We also want to improve the care of these children. While some children with possible neck injuries can be discharged home without special tests or treatment, others may require X-rays or other scans. This study will help us determine which children who have a possible neck injury require further imaging tests such as x-rays and scans.

We will collect information about your child's hospital visit and the treatment they received. This will be collected by both the emergency doctor and your child's medial notes. We would like to contact you by phone, text or email in 3-8 weeks after your visit to the Emergency Department. At this time, we would like to ask some questions about how your child has been since their visit to the Emergency Department. This will only take a couple of minutes of your time. We would like to know how your child has been and if they have had to return to the doctor or hospital for any further care for their neck injury. Please let us know if you give permission for our hospital study team to contact you, and your preferred contact method.

We will need to collect identifying information such as your child's name, date of birth and your contact details, in order to contact you. Only our local research team and *<insert local hospital>* Ethics Committee can access this identifying information.

All the other information that we collect about your child will be de-identified. This means that all personal identifiers, such as name, address and medical record number will be removed. We will combine all of the de-identified information about your child with information from other hospitals who are also participating in this study. When we write or talk about the results of this study, no-one will be able to identify you or your child.

All information collected for the SONIC study will be stored in a secure, password protected database. The database runs from the Murdoch Children's Research Institute servers, based in Melbourne, Australia. Only the research team members can access the information. There is special software that sends the survey to your mobile device if you choose to receive a text message or email. The only place your mobile number or email is stored, is on the secure study database.

Any paper documents will be kept in a locked filing cabinet at the hospital. Only SONIC researchers can access those documents. We will keep the research information for 15 years after completion of the study or until the youngest child who was involved in the study turns 25 years old. After this time, we will dispose of the information in a secure way as required by State Government policies for disposing of research information.

Involvement in the SONIC study is completely voluntary. The study doctors and study staff will only collect the information they need for this study. If you do not wish for your child to take part, please let us know and we will not collect further information and you can decide if we can use the information we have already collected for the study. If you change your mind, you are free to withdraw your consent and discontinue participation at any time. Your decision will not affect your child's medical treatment or relationship with the treating medical and nursing team at *<insert local hospital>* Hospital.
If you do decide to withdraw your child from this research project, we will not collect any further personal information about them, and you can decide if we can use the information we have already collected for the study.

Once the study has been analysed, we will make a summary of the results available for participants on a website (www.predict.org.au).

If you would like more information about the study or if you need to speak to a member of the research team please contact:

<Insert site research nurse> (research co-ordinator / assistant/nurse) and *<Insert site PI>* (site PI)
Ph: *<Insert phone number>*

You can contact the Director of Research Ethics & Governance at The Royal Children's Hospital Melbourne if you:

- have any concerns or complaints about the project
- are worried about your child's rights as a research participant
- would like to speak to someone independent of the project.

The Director can be contacted by telephone on (03) 9345 5044.
