# Supplementary material for: Prospective observational study to assess the performance accuracy of clinical decision rules in children presenting to emergency departments with possible cervical spine injuries: the Study of Neck Injuries in Children (SONIC)
Source: BMJ Open. 2025 May 2;15(5):e096294. doi: 10.1136/bmjopen-2024-096294 (PMC12049922; doi:10.1136/bmjopen-2024-096294)
Supplement: Supplementary file 2 [file bmjopen-2024-096294.reviewer_comments.pdf]

PEER REVIEW HISTORY

BMJ Open publishes all reviews undertaken for accepted manuscripts. Reviewers are asked to complete a checklist review form and are provided with free text boxes to elaborate on their assessment. These free text comments are reproduced below.

ARTICLE DETAILS

Title (Provisional)

A prospective observational study to assess the performance accuracy of clinical decision rules in children presenting to emergency departments with possible cervical spine injuries (protocol): the Study of Neck Injuries in Children (SONIC)

Authors

Phillips, Natalie; Askin, Geoffrey; Davis, Gavin A; O'Brien, Sharon; Borland, Meredith L; Williams, Amanda; Kochar, Amit; John-Denny, Blessy; Watson, Sarah; George, Shane; Davison, Michelle; Dalziel, Stuart; Tan, Eunicia; Chong, Shu-Ling; Craig, Simon; Rao, Arjun; Donath, Susan M; Selman, Chris J; Goergen, Stacy; Wilson, Catherine L; Singh, Sonia; Kuppermann, Nathan; Leonard, Julie; Babl, Franz E

VERSION 1 - REVIEW

Reviewer 1

Name Kalaiarasi, Raja

Affiliation Jawaharlal Institute of Postgraduate Medical Education, ENT

Date 13-Dec-2024

COI None

---

well written and planned study.

Can write in detail how missing data will be handled without affecting power of the study as there are many secondary objectives

---

Reviewer 2

Name Chan, Winnie Kwai Yu

Affiliation Queen Elizabeth Hospital, Hong Kong , Department of Paediatrics

Date 16-Dec-2024

COI None

---

interesting topics and good protocol

**Reviewer**	**3**
**Name**	**Munoz-Pareja, Jennifer**
**Affiliation**	**University of Miami**
**Date**	**05-Feb-2025**
**COI**	**None**

This is an excellent protocol on an understudied and highly relevant topic. If successful, it will help advance how we care for children with CSI.

VERSION 1 - AUTHOR RESPONSE

Reviewer 1:

1. *Write in detail above how missing data will be handled without affecting power of the study as there are many secondary objectives.*
   - Thank you for the comment. This study was powered based on the secondary objective where we intend to derive our own SONIC CDR. We did not power this study based on any other secondary objectives, as is common practice. However, we acknowledge that handling missing data appropriately is important to reduce potential selection/non-response bias and potentially gain more precise estimates under certain missingness mechanisms rather than reporting simply the results of a complete case analysis. Consequently, for secondary objectives that include the full eligible sample (i.e. objectives not relating to the accuracy population, where we have already commented on how missing data will be addressed in detail), we will consider using multiple imputation to handle the missing data under a plausible missingness assumptions when there is >5% missingness in key variables in the statistical model as a sensitivity analysis. We have added a paragraph under "Analysis of non-accuracy related outcomes" **[P14]** to reflect this.

Reviewers 2 & 3:

- No specific feedback to address.
